# Interplay between miRNAs and Genes Associated with Cell Proliferation in Endometrial Cancer

**DOI:** 10.3390/ijms20236011

**Published:** 2019-11-29

**Authors:** Ewelina Hermyt, Nikola Zmarzły, Beniamin Grabarek, Celina Kruszniewska-Rajs, Joanna Gola, Agnieszka Jęda-Golonka, Katarzyna Szczepanek, Urszula Mazurek, Andrzej Witek

**Affiliations:** 1 Department of Gynecology and Obstetrics, Faculty of Medical Sciences in Katowice, Medical University of Silesia in Katowice, Medyków 14, 40-752 Katowice, Poland; ewelina.hermyt@gmail.com (E.H.); ajeda@mp.pl (A.J.-G.); eukariot@op.pl (K.S.); awitek@sum.edu.pl (A.W.); 2 Department of Molecular Biology, Faculty of Pharmaceutical Sciences in Sosnowiec, Medical University of Silesia in Katowice, Jedności 8, 41-200 Sosnowiec, Poland; bgrabarek7@gmail.com (B.G.); ckruszniewska@sum.edu.pl (C.K.-R.); jgola@sum.edu.pl (J.G.); 3 Department of Histology, Faculty of Medicine, University of Technology, Park Hutniczy 3-5, 41-800 Zabrze, Poland; 4 Center of Oncology, M. Sklodowska-Curie Memorial Institute, Cracow Branch, Garncarska 11, 31-115 Kraków, Poland; 5 Jozef Tyszkiewicz Higher School in Bielsko-Biała, Nadbrzeżna 12, 43-300 Bielsko-Biała, Poland; urszula.mazurek@tyszkiewicz.edu.pl

**Keywords:** endometrial cancer, proliferation, miRNA, microarray

## Abstract

Endometrial cancer develops as a result of abnormal cell growth associated with uncontrolled cell proliferation, excessive activation of signaling pathways and miRNA activity. The aim of this study was to determine the expression profile of genes associated with cell proliferation and to assess which miRNAs can participate in the regulation of their expression. The study enrolled 40 patients with endometrial cancer and 10 patients without neoplastic changes. The expression profile of genes associated with cell proliferation and the expression profile of miRNAs were assessed using microarrays. RT-qPCR was performed to validate mRNA microarray results. The mirTAR tool was used to identify miRNAs that regulate the activity of genes associated with cell proliferation. Decreased expression of *IGF1* and *MYLK*, as well as *SOD2* overexpression, were observed in endometrial cancer using both mRNA microarrays and RT-qPCR. Microarray analysis showed low levels of *NES* and *PRKCA*, but this was only partially validated using RT-qPCR. Reduced activity of *MYLK* may be caused by increased miR-200c, miR-155 and miR-200b expression. Cell proliferation is disturbed in endometrial cancer, which may be associated with an overexpression of miR-200a, miR-200c, and miR-155, making it a potential diagnostic marker.

## 1. Introduction

Endometrial cancer develops as a result of abnormal growth of the cells, which in consequence acquire the ability to migrate and invade surrounding tissues. The highest incidence concerns women in the peri- and postmenopausal period [1]. It is possible to distinguish two types of endometrial cancer according to clinical–pathological and molecular characteristics. Type I (estrogen-dependent) accounts for 80% of endometrial cancer cases, and progesterone and estrogen receptors are expressed in cancer tissue. The formation of type II cancer is not dependent on estrogenic stimulation, and progesterone and estrogen receptors usually are not expressed [1,2,3]. Endometrial cancer can be also divided according to the degree of histological differentiation: G1 (≤5% solid growth pattern), G2 (6–50% solid growth pattern), G3 (>50% solid growth pattern) [4].

Carcinogenesis is associated with the disruption of cell cycle regulation, which leads to uncontrolled cell proliferation. It is also caused by excessive activation of signaling pathways involved in stimulating cell growth. In normal tissue, cell growth and development are regulated to prevent abnormal proliferation. The occurrence of mutations and epigenetic mechanisms, including miRNAs, is observed during the neoplastic process. It leads to increased survival and growth of cancer cells by acquiring the ability to invade and metastasize [5]. MicroRNA (miRNA) molecules are small, non-coding RNAs responsible for post-transcriptional regulation of gene expression [6]. Complete complementarity between mRNA and miRNA causes transcript degradation, while partial complementarity inhibits its translation [7]. Changes in miRNA activity in cancer lead to abnormal cell proliferation, apoptosis and angiogenesis. As a result, tumor progression is observed [8].

The aim of this study was to determine the expression profile of genes associated with cell proliferation and to assess which miRNAs can participate in the regulation of their expression.

## 2. Results

### 2.1. mRNA Microarrays

The one-way ANOVA with Benjamini–Hochberg correction showed that among the 321 mRNA-representing genes associated with cell proliferation, 63 mRNAs were differentially expressed in endometrial cancer compared to the control at *p* < 0.05. A Tukey’s post-hoc test indicated that the number of mRNAs differentiating each endometrial cancer grade from the control was as follows: G1 vs. control, 18; G2 vs. control, 44; and G3 vs. control, 23 (*p* < 0.05) (Table 1).

Table 2 shows mRNAs that specifically differentiate each grade of endometrial cancer from the control at *p* < 0.05 and fold-change (FC) cut-off >2 or <−2.

It was observed that according to the criteria established in this work, the expression of *PRKCA*, *NES*, *FGF9*, *MYLK*, *IGF1*, *CACNA1I*, *ESR1*, and *SOD2* significantly changed in G2 endometrial cancer. In turn, statistically significant changes in *MAP2K3* activity were reported in G3 cancer (Table 2).

### 2.2. miRNA Microarrays

An ANOVA test showed that among the 1105 miRNAs specific in humans, 30 miRNAs have shown significant differences in expression ofendometrial cancer compared to the control (*p* < 0.05 and FC cut-off >2 or <−2). A Tukey’s post-hoc test indicated that the number of differentiating miRNAs was 2 in G1 and 28 in G2 endometrial cancer. In the next step, using the mirTAR tool, it was assessed which of these miRNAs are involved in the activity regulation of genes associated with cell proliferation that differentiate endometrial cancer from the control (Table 3).

The analysis showed that among the 30 miRNAs differentiating endometrial cancer from the control, 17 miRNAs may participate in the expression regulation of genes associated with cell proliferation. Decreased *FGF9* activity may be the result of increased miR-182 expression in G2 endometrial cancer. Overexpression of miR-200c, miR-155 and miR-200b may lead to silencing of *MYLK*. In addition, low levels of *IGF1* may be caused by miR-625, let-7f, miR-331-3p, let-7g, and let-7a. MiR-625 and miR-331-3p together with miR-15b and miR-200a may participate in reducing *PRKCA* expression. The simultaneous increase in *SOD2* and miR-331-3p level may suggest that the regulation of *SOD2* activity in endometrial cancer occurs at the translational level (Table 3).

### 2.3. RT-qPCR

A Shapiro–Wilk test showed that the RT-qPCR results did not meet the normal distribution assumptions. Changes in the expression of examined genes are presented as median (Me), lower (Q1) and upper (G3) quartile. The Kruskal–Wallis and Dunn’s tests showed statistically significant differences in the expression of *NES*, *SOD2*, *MYLK* and *IGF1* (*p* < 0.05; Table 4).

Considering the FC value and median, the same direction of change in *IGF1*, *SOD2* and *MYLK* expression was observed in both mRNA microarray and RT-qPCR analysis. In the case of *NES*, slight differences were noted in G2 endometrial cancer, and for *PRKCA* in G1 and G3 samples (Table 2; Table 4).

## 3. Discussion

It is believed that carcinogenesis is the result of an abnormal proliferation rate. It is caused by the disruption of cell cycle regulation and excessive activation of signaling pathways involved in stimulating cell growth. In normal tissue, cell growth and development are subject to numerous regulations to prevent uncontrolled proliferation. During the neoplastic process, these mechanisms do not work properly due to the occurrence of mutations and miRNAs activity [5,8]. As a result, the survival of cancer cells increases and they acquire the ability to invade and metastasize [5]. Restoration of normal proliferation and induction of apoptosis can be a promising therapeutic target for cancer, including endometrial cancer, as well as other pathologies, such as endometriosis [9,10].

IGF-1 belongs to the family of factors responsible for the regulation of cell growth, proliferation, differentiation, apoptosis and promotion of cell motility [9]. A correlation between circulating IGF-1 and the risk of developing breast cancer, colorectal cancer, lung cancer and prostate cancer has been described [11,12]. Our study showed a significant reduction in *IGF1* expression in endometrial cancer compared to the control, which may be due to increased let-7a, let-7f, let-7g, miR-625 and miR-331-3p activity. Guo et al. found that let-7a inhibits proliferation, migration and invasion of cervical cancer cells [13]. Tang et al. came to similar conclusions in a study on gastric cancer [14]. In the case of miR-331-3p, its reduced expression promoted cell proliferation of colorectal cancer [15] and epithelial ovarian carcinoma [16]. Interestingly, Chen et al. observed that downregulation of this miRNA leads to the inhibition of prostate cancer cell proliferation and metastasis associated with epithelial-mesenchymal transition (EMT) [17].

Our study has shown that miR-331-3p may also be involved in the regulation of *SOD2* activity. The expression of SOD2 is altered in various types of cancers, including squamous cell carcinoma, lymphoma, leukemia, sarcomas, colon cancer, breast cancer, esophageal cancer, pancreatic cancer, liver cancer, lung cancer and central nervous system cancers [18]. Chang et al. observed that an increase in SOD2 level promoted distant metastases and reduced overall survival and disease-free survival [19]. Md Fuzi et al. showed SOD2 overexpression in endometrial cancer and suggested it as a potential therapeutic target [20]. These results are confirmed by our study. Moreover, the simultaneous increase in *SOD2* and miR-331-3p levels may indicate that miRNA-mediated gene expression regulation occurs at the translation level, but more research is needed.

MYLK is an enzyme that participates in processes associated with the activation of myosin, such as cell adhesion, division, migration and invasion. It has also been reported that MYLK promotes progression and metastasis of hepatocellular carcinoma [21] and gastric cancer [22]. On the other hand, MYLK expression at both mRNA and protein levels was significantly reduced in non-small-cell lung cancer compared to healthy lung tissue, which may result in increased mutagenesis that promotes cell proliferation and drives carcinogenesis [23]. *MYLK* activity can be regulated by miR-200c and miR-155, which were overexpressed in our study. Researchers observed that a high level of miR-200c in epithelial ovarian cancer was associated with lymph node metastasis, advanced cancer stage and poor overall survival. It has also been shown that the increased plasma levels of miR-200c and miR-200a in patients with breast cancer may indicate metastasis up to two years before clinical diagnosis [24]. In the case of miR-155, a decrease in its expression was observed in colorectal cancer cells, which led to inhibition of cell proliferation, induction of cell cycle arrest and apoptosis [25]. In turn, Qu et al. noted overexpression of miR-155 in gastric cancer, which was associated with the promotion of tumor cell proliferation and migration [26]. Gao et al. found that high miR-155 levels in colon cancer enhanced drug resistance, which could be a promising therapeutic target [27].

PRKCA belongs to the serine/threonine protein kinase family, which is responsible for cell survival, proliferation, apoptosis and migration [28]. Studies in mice showed that a PRKCA knockout led to the spontaneous formation of intestinal cancer [29]. In turn, Ways et al. demonstrated that overexpression of *PRKCA* in MCF-7 breast cancer cells increases proliferation rate and tumorigenicity in nude mice [30]. Similarly, Tonetti et al. noted that increased *PRKCA* expression in T47-D breast cancer cells occurs along with decreased ER function [31] and shows hormone-independent growth that cannot be inhibited by tamoxifen [32]. In our study, the *PRKCA* level was reduced, which may be associated with overexpression of miR-625, miR-331-3p, miR-15b and miR-200a. It was observed that low levels of miR-15b in gliomas were associated with poor overall survival [33], increased proliferation, cell invasion and migration [34]. On the other hand, inhibition of miR-15b activity leads to reduced migration and metastasis in colorectal cancer [35]. Liu et al. also observed overexpression of miR-15b in hepatocellular carcinoma cell lines and serum from patients. Interestingly, a decrease in the level of serum miRNA was noted after surgery [36]. In the case of miR-200a, its decreased expression in colorectal cancer promotes poor prognosis [37]. Gao et al. observed a low level of miR-200a in neuroblastoma [38], while Suo et al. reported its overexpression in ovarian cancer [39].

Endometrial cancer treatment often involves surgical removal of the uterus, ovaries, uterine tubes, and pelvic and paraaortic lymph nodes [40]. In the case of reproductive-aged women, fertility-sparing treatment is possible, however, it requires further standardization and development in order to provide the patient with access to various solutions [41,42]. It is important to look for complementary molecular markers that allow for an earlier and more precise diagnosis, as it can provide more treatment options and allow for the development of strategies that will be as non-invasive as possible for the patient. This, in turn, will allow a quick recovery and minimize the chance of potential complications [43,44]. Therapy selection and management protocol is closely related to the type, stage and grade of endometrial cancer, however, it is still not fully accurate. The Cancer Genome Atlas Research Network (TCGA) proposed an additional division of endometrial cancer into four molecular subtypes: POLE ultramutated, microsatellite instability hypermutated, copy number low, and copy number high, which emphasizes the high heterogeneity of this cancer [42]. Therefore, the classification of endometrial cancer taking into account changes at the molecular level is very important. Moreover, it should be remembered that these changes are ahead of phenotypic changes, which may allow for more precise diagnostics and therapy [45].

In this work, mRNA and miRNA microarray techniques were used, which allowed for obtaining a large amount of data regarding the transcriptome of endometrial cancer. The validation of the expression profile of genes associated with proliferation using RT-qPCR is the strength of this study. Partial confirmation of results may, however, be the result of a different number of patients in analyzes, as well as individual variability and tumor heterogeneity. In the next stage of the study, it would be important to carry out the analysis at the protein level, which would allow determination of expression changes at different levels of genetic information flow. In addition, a comprehensive assessment of the regulatory effect of miRNAs on the activity of the genes under study would be possible.

Studies carried out as part of this work showed deregulation of proliferation in endometrial cancer, which may be associated with reduced levels of *IGF1*, *MYLK* and overexpression of *SOD2*. The levels of miR-200a, miR-200c and miR-155, probably involved in the regulation of *MYLK* activity, were increased in endometrial cancer compared to the control. Their overexpression may promote uncontrolled proliferation, which may be associated with tumor progression, making them potential diagnostic markers for endometrial cancer.

## 4. Materials and Methods

The study enrolled 50 patients who underwent hysterectomy: 40 with endometrial cancer (study group) and 10 patients without neoplastic changes during routine gynecological examinations (control group). Exclusion criteria from the study group included endometrial hyperplasia with or without atypia, diagnosis of cancer other than endometrial adenocarcinoma, extreme obesity (body mass index >40) and use of hormone replacement therapy 5 years prior to the surgery. The histopathological assessment of endometrial tissue samples allowed us to divide the study group according to the degree of histological differentiation: G1 (well-differentiated), 10; G2 (moderately differentiated), 20; and G3 (poorly differentiated), 10 cases. Collected samples were stored in RNAlater™ (Sigma-Aldrich, Saint Louis, MO, USA) according to the manufacturer’s protocol. This study was approved by the Bioethical Committee of the Medical University of Silesia (25 October 2016, Sosnowiec, Poland; no. KNW/0022/KB1/130/16). Written informed consent was obtained from all of the patients recruited.

The extraction of total RNA was performed using the TRIzol^®^ reagent (Invitrogen; Thermo Fisher Scientific, Inc., Waltham, MA, USA) according to the manufacturer’s protocol. The expression profile of genes associated with cell proliferation was evaluated using a microarray technique (HG-U133A; Affymetrix, Santa Clara, CA, USA) in 27 samples (control, 3; G1, 7; G2, 11; and G3, 6). The first step included the use of 8 µg of RNA as a template in order to synthesize cDNA using SuperScript Choice System (Invitrogen Technologies, Carlsbad, CA, USA). Biotinylated cRNA was then synthesized with the use of BioArray HighYield RNA Transcript Labeling Kit (Enzo Life Sciences, Farmingdale, NY, USA). Obtained cRNA was purified with RNeasy Mini Kit (Qiagen GmbH, Hilden, Germany). The next step included fragmentation of the biotin-labeled cRNA performed with the Sample Cleanup Module Kit (Qiagen GmbH, Hilden, Germany). cRNA was stained with streptavidin–phycoerythrin after it hybridized to the HG-U133A microarray. Gene Array Scanner G2500A (Agilent Technologies, Santa Clara, CA, USA) was used to measure fluorescence signals.

The expression profile of miRNAs was determined in 11 endometrial tissue samples (control, 4; G1, 3; G2, 4). First, RNA was labeled with biotin with the FlashTag Biotin HSR RNA Labeling Kit (Affymetrix, Santa Clara, CA, USA). The ELOS QC assay was used to verify the labeling efficiency. In the next step, the labeled molecules were hybridized to a miRNA 2.0 microarray (Affymetrix, Santa Clara, CA, USA), containing 15,644 probes in total, including 1105 specific for human miRNAs. After washing and staining with Hybridization Wash and Stain Kit (Affymetrix, Santa Clara, CA, USA) and Fluidics Station 450 (Affymetrix, Santa Clara, CA, USA), microarrays were scanned with GeneChip Scanner 3000 7G (Affymetrix, Santa Clara, CA, USA) and the received signals were read using the Affymetrix^®^ GeneChip^®^ Command Console^®^ Software (AGCC) (Affymetrix, Santa Clara, CA, USA).

The results of the mRNA microarray analysis were validated by RT-qPCR. The reaction was carried out using SensiFAST SYBR No-ROX One-Step Kit (Bioline, London, UK) and OpticonTM DNA Engine Sequence Detector (MJ Research Inc., Watertown, MA, USA), according to the manufacturer’s protocol. It was performed with the use of the following primers: *IGF1* (forward: 5′ CCCAGAAGGAAGTACATTTG 3′, reverse: 5′ GTTTAACAGGTAACTCGTGC 3′), *MYLK* (forward: 5′ AGAATCTGAAGATGTGTCCC 3′, reverse: 5′ ATCTTGCAGTCAAATCTAGC 3′), *NES* (forward: 5′ ATGGAGACGTCGCTG 3′, reverse: 5′ ACAGCCAGCTGGAAC 3′), *PRKCA* (forward: 5′ CCAAAGTGTGTGGCAAAG 3′, reverse: 5′ TCAGACTGGTCTATGTTAGC 3′), *SOD2* (forward: 5′ ATCATACCCTAATGATCCCAG 3′, reverse: 5′ AGGACCTTATAGGGTTTTCAG 3′). All 50 endometrial tissues samples were used during this step of molecular analysis.

Statistical analysis of the results obtained in this mRNA microarray experiment was performed using GeneSpring GX 13.0 software (Agilent Technologies, Inc., Santa Clara, CA, USA) and PL-Grid Infrastructure (http://www.plgrid.pl/en). Comparative analysis was carried out for 321 mRNA-encoding proteins associated with cell proliferation selected based on the literature data and the Affymetrix NetAffx™ Analysis Center database (http://www.affymetrix.com/analysis/index.affx; accessed on 17 October 2019). A one-way ANOVA with Benjamini–Hochberg correction and a Tukey’s post-hoc test were carried out. The microarray dataset has been deposited in the Gene Expression Omnibus (GEO) Database at the National Center for Biotechnology Information (NCBI) under the accession GSE115810. In the case of the miRNA microarrays, Transcriptome Analysis Console 4.0 (Affymetrix, Santa Clara, CA, USA) was used to perform one-way ANOVA and Tukey’s post-hoc tests. Then, the mirTAR tool (http://mirtar.mbc.nctu.edu.tw/human/predictionIndex.php; accessed on 17 October 2019) was used to identify the miRNAs involved in the expression regulation of genes associated with cell proliferation. Statistical analysis of RT-qPCR results was carried out with the Statistica 13.1 PL software (StatSoft, Tulsa, OK, USA). To determine whether the data met normal distribution assumptions, a Shapiro–Wilk test was performed. The lack of normal distribution allowed for analysis based on nonparametric tests (Kruskal–Wallis and Dunn’s post-hoc tests).

## Figures and Tables

**Table 1:** The number of mRNAs that are differentially expressed in endometrial tissue samples.

Group	C	G1	G2	G3
C	63	18 ^1^	44 ^1^	23 ^1^
G1	45	63	17 ^2^	27 ^2^
G2	19	46	63	18 ^3^
G3	40	36	45	63

C, control; G, grade of endometrial cancer. ^1^ G1, G2, G3 vs. C at *p* < 0.05. ^2^ G2, G3 vs. G1 at *p* < 0.05. ^3^ G3 vs. G2 at *p* < 0.05.

**Table 2:** List of transcripts associated with cell proliferation differentiating endometrial cancer from the control. *p* < 0.05 and FC > 2 or FC <−2.

Groups Compared	ID	Gene	*p*-Value	FC	Expression
G2 vs. C	213093_at	*PRKCA*	0.0051	−2.7616084	decreased
	218678_at	*NES*	0.0007	−3.5484042	decreased
	206404_at	*FGF9*	0.0020	−3.770779	decreased
	202555_s_at	*MYLK*	0.0001	−7.969888	decreased
	209540_at	*IGF1*	0.0001	−11.902487	decreased
	208299_at	*CACNA1I*	0.0042	2.0332716	increased
	211234_x_at	*ESR1*	0.0036	2.0671976	increased
	221477_s_at	*SOD2*	0.0056	3.41224	increased
	215223_s_at	*SOD2*	0.0079	3.9286928	increased
G3 vs. C	215498_s_at	*MAP2K3*	0.0000	2.0928204	increased

ID, number of the probe; FC, fold-change; C, control; G, grade of endometrial cancer.

**Table 3:** List of genes associated with proliferation, whose activity may be regulated by miRNAs in endometrial cancer, determined by mRNA microarrays and mirTAR tool.

Gene	Expression	miRNA	*p*-Value	FC	Expression
*FGF9*	decreased	miR-182	0.0053	49.39	increased
*SOD2*	increased	miR-331-3p	0.0178	5.54	increased
*NES*	decreased	miR-432	0.0159	−11.28	decreased
*MYLK*	decreased	miR-432	0.0159	−11.28	decreased
		miR-200c	0.0399	4.36	increased
		miR-155	0.0398	9.62	increased
		miR-200b	0.0010	73.52	increased
*CACNA1I*	increased	miR-1296	0.0158	−4.81	decreased
		miR-483-5p	0.0441	−10.24	decreased
		miR-432	0.0159	−11.28	decreased
		miR-874	0.0078	5.69	increased
		miR-10a	0.0027	37.2	increased
*IGF1*	decreased	miR-432	0.0159	−11.28	decreased
		miR-625	0.0235	3.21	increased
		let-7f	0.0206	3.7	increased
		miR-331-3p	0.0178	5.54	increased
		let-7g	0.0214	13.01	increased
		let-7a	0.0027	37.2	increased
*ESR1*	increased	miR-370	0.0444	−10.91	decreased
		miR-432	0.0159	−11.28	decreased
		miR-625	0.0235	3.21	increased
		miR-15b	0.0173	3.71	increased
		miR-331-3p	0.0178	5.54	increased
		miR-874	0.0078	5.69	increased
		miR-10a	0.0027	37.2	increased
*PRKCA*	decreased	miR-1296	0.0158	−4.81	decreased
		miR-483-5p	0.0441	−10.24	decreased
		miR-370	0.0444	−10.91	decreased
		miR-432	0.0159	−11.28	decreased
		miR-625	0.0235	3.21	increased
		miR-15b	0.0173	3.71	increased
		miR-331-3p	0.0178	5.54	increased
		miR-200a	0.0229	8.32	increased
*MAP2K3*	increased	miR-483-5p	0.0441	−10.24	decreased
		miR-370	0.0444	−10.91	decreased
		miR-15b	0.0173	3.71	increased
		miR-874	0.0078	5.69	increased

ID, number of the probe; FC, fold-change; C, control; G, grade of endometrial cancer.

**Table 4:** Values of descriptive statistics, Kruskal–Wallis and post-hoc tests in endometrial cancer and control (*p* < 0.05).

Gene	Group	mRNA Copies/μg Total RNA			Kruskal-Wallis Test	Post-hoc Test
		Me	Q1	Q3		
*IGF1*	C	76,580	37,330	241,900	0.037	G3 vs. C, *p* = 0.033643
	G1	37,965	11,370	45,180		
	G2	29,140	10,390	55,660		
	G3	10,900	9437	23,440		
*SOD2*	C	303,400	250,100	368,281	0.0252	G2 vs. C, *p* = 0.021202 G3 vs. C, *p* = 0.040698
	G1	749,800	510,900	1,942,000		
	G2	1,031,000	485,400	2,839,000		
	G3	1,484,000	576,700	2,375,000		
*MYLK*	C	98,365	56,266	217,400	0.0368	G1 vs. C, *p* = 0.058917 G2 vs. C, *p* = 0.059353 G3 vs. C, *p* = 0.362364
	G1	19,455	12,440	41,290		
	G2	25,200	12,370	59,660		
	G3	30,350	13,710	60,400		
*NES*	C	183	146	220	0.0001	G2 vs. G1, *p* = 0.044928 G3 vs. G2, *p* = 0.000025
	G1	123	68	161		
	G2	570	292	818		
	G3	26	0	101		
*PRKCA*	C	11,190	6378	13,630	0.9491	NS
	G1	13,310	8564	17,550		
	G2	9517	4890	23,040		
	G3	13,585	4671	20,890		

Me, median; Q1, lower quartile; Q3, upper quartile; C, control; G, grade of endometrial cancer; NS, not significant.

## Abbreviations

IGF1	insulin-like growth factor 1
MYLK	myosin light chain kinase
NES	nestin
PRKCA	protein kinase C alpha
SOD2	superoxide dismutase 2
FGF9	fibroblast growth factor 9
CACNA1I	calcium voltage-gated channel subunit alpha1 I
ESR1	estrogen receptor 1
MAP2K3	mitogen-activated protein kinase kinase 3
FC	fold-change
